# *Borrelia burgdorferi* Elicited-IL-10 Suppresses the Production of Inflammatory Mediators, Phagocytosis, and Expression of Co-Stimulatory Receptors by Murine Macrophages and/or Dendritic Cells

**DOI:** 10.1371/journal.pone.0084980

**Published:** 2013-12-19

**Authors:** Yutein Chung, Nan Zhang, R. Mark Wooten

**Affiliations:** Department of Medical Microbiology and Immunology, University of Toledo College of Medicine, Toledo, Ohio, United States of America; University of Kentucky College of Medicine, United States of America

## Abstract

Borrelia burgdorferi (Bb) is a tick-borne spirochete that is the causative agent for Lyme disease. Our previous studies indicate that virulent Bb can potently enhance IL-10 production by macrophages (MØs) and that blocking IL-10 production significantly enhances bacterial clearance. We hypothesize that skin-associated APC types, such as MØs and dendritic cells (DCs) are potent producers of IL-10 in response to Bb, which may act in autocrine fashion to suppress APC responses critical for efficient Bb clearance. Our goal is to delineate which APC immune functions are dysregulated by Bb-elicited IL-10 using a murine model of Lyme disease. Our *in vitro* studies indicated that both APCs rapidly produce IL-10 upon exposure to Bb, that these levels inversely correlate with the production of many Lyme-relevant proinflammatory cytokines and chemokines, and that APCs derived from IL-10^-/-^ mice produced greater amounts of these proinflammatory mediators than wild-type APCs. Phagocytosis assays determined that Bb-elicited IL-10 levels can diminish Bb uptake and trafficking by MØs, suppresses ROS production, but does not affect NO production; Bb-elicited IL-10 had little effect on phagocytosis, ROS, and NO production by DCs. In general, Bb exposure caused little-to-no upregulation of several critical surface co-stimulatory markers by MØs and DCs, however eliminating Bb-elicited IL-10 allowed a significant upregulation in many of these co-stimulatory receptors. These data indicate that IL-10 elicited from Bb-stimulated MØs and DCs results in decreased production of proinflammatory mediators and co-stimulatory molecules, and suppress phagocytosis-associated events that are important for mediating both innate and adaptive immune responses by APCs.

## Introduction

Lyme disease is caused by the tick-borne spirochetal bacterium Borrelia burgdorferi (Bb)[1]. After deposition into the skin of susceptible hosts, this pathogen can rapidly migrate through skin tissues to infect and persist within many different tissues [2]. If diagnosed and treated with appropriate antibiotics in a timely manner, these bacteria are usually cleared from most patients without lasting symptoms. However, in the absence of antibiotic treatment, these pathogens can persist for months to years in a wide range of host tissues. Persistent bacteria can periodically “re-emerge” in those tissues to elicit inflammatory responses that cause the widely varying sequellae associated with Lyme disease; this is most commonly observed as inflammation in large joints, nervous and neurologic abnormalities, and cardiac-associated disorders. Notably, immunocompetent hosts are able to produce potent innate and adaptive immune responses against Bb during the course of infection. *In vitro* studies have shown that macrophages and neutrophils can phagocytose and kill Bb quite efficiently, particularly if Bb-specific antibodies are present [3-5]. While the adaptive immune response to Bb is somewhat delayed, the antibodies produced during this response are able to bind Bb and mediate killing *in vitro* and passive transfer of these antisera into naïve mice are able to prevent subsequent infection by similar Bb strains [6,7]. However, even though Bb appears to disseminate and persist largely within the extracellular tissues, where they should be readily accessible to both cellular and soluble immune mediators, these innate and adaptive immune effectors are unable to effectively clear these pathogens. Thus there is great interest in identifying the mechanisms that allow Bb to evade these immune responses. 

Appropriate innate immune responses appear to be particularly critical in controlling the development of Lyme disease [1,8]. The Bb genome encodes ≥127 different lipoproteins (e.g. ~8% of all open reading frames), many of which are believed to be important for their ability to rapidly adapt and persist within tick and vertebrate hosts [2,9-13]. While these numerous lipoproteins likely provide different biological functions, all appear to possess similar triacyl modifications at the amino terminal that promote their trafficking and insertion into the Bb outer membrane [14,15]. Murine studies have shown that innate receptors, most notably CD14 and toll-like receptor 2 (TLR2), can recognize the common triacyl motif on these lipoproteins and initiate inflammatory responses via MyD88-dependent pathways [16-20]. These TLR2-mediated signaling pathways are critical for macrophage (MØ) activation by Bb lipoproteins, leading to more efficient intracellular trafficking of Bb, and the production of a wide range of inflammatory mediators believed important for promoting Bb clearance [21,22]. It is likely that these pathways are also used in a number of other immune and non-immune cell types that are also directly activated by Bb lipoproteins, including dendritic cells (DCs) [23,24], neutrophils [25], mast cells [26], B cells [27], and endothelial cells [28,29]. Importantly, TLR2-deficient (TLR2^-/-^) mice infected with Bb possess up to 100-fold higher bacterial loads than wild-type mice in different tissues at both early and late times post-infection, even though these TLR2^-/-^ mice produced Bb-specific antibodies at similar levels and of similar Bb-antigen specificity as infected wild-type mice [21,30]. These findings highlight the ability of these spirochetes to efficiently evade the adaptive immune responses and the importance of innate responses in controlling Bb levels during all stages of infection.

The anti-inflammatory cytokine IL-10 is known to play a significant role in the development of Lyme disease [31]. IL-10 can be produced by a number of different leukocyte and non-immune cell types in response to various stimuli, but the most potent production is usually associated with myeloid cells and certain T cell subsets [32,33]. The timely elicitation of IL-10 in tissues is critical for the dampening and resolution of inflammatory responses, as extended immune activation would result in inadvertent damage to host tissues. Patients with deficiencies in IL-10 often develop disease manifestations related to uncontrolled inflammatory conditions, such as inflammatory bowel disorder, rheumatoid arthritis, and several autoimmune diseases [34], thus confirming the important and central role this cytokine plays in modulating inflammatory signaling. A number of *in vitro* studies have reported that monocytes/macrophages exposed to intact Bb or a standard Bb recombinant lipoprotein (OspA) exhibit a potent IL-10 response [35-37]. Primary MØs derived from mouse lines known to develop either severe (C3H/HeN; C3H) or mild (C57BL/6; B6) Lyme disease during Bb infection showed that B6 MØs display a significantly greater IL-10 response to OspA lipoprotein than C3H MØs, and that addition of physiologic levels of IL-10 to OspA-stimulated C3H MØs reduced their production of proinflammatory cytokines to levels observed in B6 MØs, suggesting these differences in IL-10 production might influence the severity of Lyme disease [37,38]. Infection studies using B6 mice lacking a functional IL-10 gene (IL-10^-/-^) showed these animals developed increased ankle swelling and arthritis severity compared to wild-type controls, even though the IL-10^-/-^ mice maintained ~10-fold less bacteria in affected tissues [37]. Gene microarray analyses of infected joint tissues from B6-IL-10^-/-^ mice determined that the gene expression patterns were more similar to those for infected C3H mice displaying severe disease, as opposed to infected B6 mice that develop mild disease [39], and blocking these IL-10 mediated responses reduced the infiltration of different immune cells whose presence is associated with increased arthritis severity [40], suggesting an important role for IL-10 in mediating Lyme-associated pathology. Notably, infected IL-10^-/-^ mice possess significantly reduced levels of Bb loads in ankle and other tissues compared to B6 mice [37]. Although infected IL-10^-/-^ mice do possess higher Bb-specific antibody levels than B6 mice, this enhanced ability to clear Bb infection appears to occur largely via effects on the innate immune response [41]. This IL-10 response is rapid, as substantial production is seen by macrophages *in vitro* within 4-6h after co-culture and significant increases in IL-10 transcript levels are seen in skin tissues within 24h post-infection with a physiological dose of Bb [41,42]. The IL-10^-/-^ mouse is currently the only infection model where manipulation of a single soluble immune mediator can significantly enhance Bb clearance in multiple target tissues, thus there is great interest in better understanding the mechanisms affected during IL-10 suppression of Bb clearance.

MØs and DCs are believed to be important for initiating the immune response to Bb infection, due to their proximity in the skin, ability to phagocytose and be activated by Bb, ability to initiate inflammatory responses, and ability to act as antigen-presenting cells (APCs) in initiating adaptive immunity [43,44]. Both of these APCs are known to be major producers of IL-10 in response to different stimuli, and are also known to downregulate several of their immune mechanisms in response to IL-10 [33]. Based on the ability of Bb to elicit strong IL-10 production, we hypothesize that MØs and DCs are major producers of IL-10 in response to Bb, which can then act in an autocrine fashion to suppress immune activities that are critical for the control of Bb infection. The goal of these studies is to determine the relative abilities of MØs and DCs to produce IL-10 in response to Bb, and delineate whether these IL-10 levels affect their abilities to suppress immune mechanisms that are critical for mediating both innate and adaptive immune responses to Bb. 

## Materials and Methods

### *B. burgdorferi* growth and viability

A clonal N40 isolate of Bb [45] was provided by Steve Barthold (University of California, Davis), and were maintained in BSK-II medium [46] supplemented with 6% rabbit serum (Pel-Freez Biologicals)(BSK-II). A B31-5A-14 Bb isolate engineered to express eGFP from the borrelial *erpAB* promoter [47] was provided by Brian Stevenson (University of Kentucky, Lexington) and was maintained in BSK-II containing 200μg/ml kanamycin. For all experiments, both strains were grown at 33°C for 3-5 days before enumerating by direct counting using a Petroff-Hauser chamber and dark field microscopy. All *in vitro* experiments were performed using bacteria that are between passages 4-7 *ex vivo*. 

### Animal usage

C57BL/6 (B6) wild-type mice were purchased from Charles River Laboratories (NCI-Frederick). B6.129P2-IL-10*tm1Cgn*/J mice lacking a functional IL-10 gene (IL-10^-/-^) were purchased from The Jackson Laboratory. All animals were housed in the Department of Lab Animal Research at the University of Toledo Health Science Campus according to National Institute of Health guidelines for the care and use of laboratory animals. All usage protocols were reviewed and approved by the Institutional Animal Care and Usage Committee (IACUC) at the University of Toledo. 

#### 1. Expansion of primary MØ and DC for *in vitro* analyses

**Figure 1:**
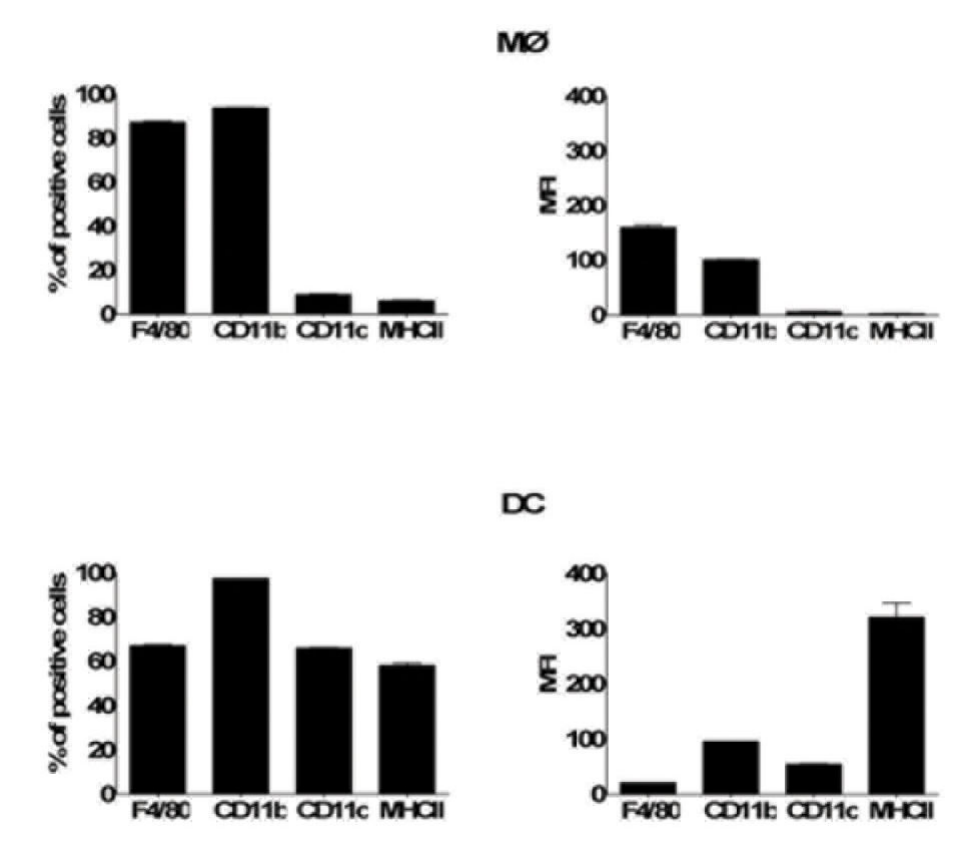
Surface markers on naïve MØs and DCs. Bone marrow-derived naïve MØs and DCs were collected prior to addition of Bb, stained with antibodies specific for the indicated surface markers, and analyzed by flow cytometry.

Bone marrow-derived primary MØs were prepared as previously described [41]. Briefly, dissociated marrow tissues from the limb bones of B6 or IL-10^-/-^ mice were isolated and cultured for 6 days in RPMI media containing 30% L929 cell supernatants and 10% FBS. Adherent cells were collected, enumerated, and re-seeded onto tissue culture plates as indicated for the particular assay. Bone marrow-derived primary DCs were prepared as previously described [48]. Briefly, dissociated marrow tissues were cultured for 5 days in RPMI media containing 10ng/ml recombinant GM-CSF (R&D Systems) and 10% FBS, then the non-adherent cells were collected, enumerated, and seeded onto tissue culture plates as indicated for the particular assay in medium lacking GM-CSF. Before using in experiments, the phenotype of these expanded MØ and DC populations was examined by flow cytometry (FACSCalibur™, BD Biosciences) using fluorescent antibodies (BD Biosciences-Pharmingen) against murine CD11b, CD11c, F4/80, and MHC class II (Figure 1). In general, naïve MØs were high in CD11b and F4/80, while low on MHC class II and CD11c, whereas naïve DCs were high in CD11b, CD11c and MHC class II (Figure 1). 

### *In vitro* ELISA analyses

Naïve MØs and DCs were seeded on 24-well tissue-culture treated plates at 4 x 10^5^ cells per well in 0.5 ml of RPMI containing 10% FBS and 20% BSK-II medium (RPMI-B) and allowed to adhere overnight. Bb were added at a multiplicity of infection (MOI) of 10, centrifuged at 300 x g for 5 min to facilitate contact, and incubated at 37°C in 5% CO_2_. For some experiments, co-culture was performed in the presence of an IL-10-blocking antibody (3μg/ml; BD Biosciences), thus allowing delineation of effects due to IL-10 versus those inherent to the IL-10-deficient background of the IL-10^-/-^ APCs. At the indicated times post-infection, supernatants were collected and either frozen for later use or immediately assessed for cytokine content by sandwich ELISA using our previously described methods [37,41]. ELISA plates were prepared using paired mAbs and cytokine standards (BD Biosciences), and bound cytokines were visualized using avidin-HRP (Vector Laboratories) and quantified at 490nm using a Versamax® (Molecular Devices) 96-well plate reader. All experimental conditions were performed in triplicate and repeated in at least 4 separate experiments.

### Quantitative RT-PCR

Naïve MØs and DCs were seeded on 12-well tissue-culture treated plates at 1 x 10^6^ cells per well in 1ml of RPMI-B and allowed to adhere overnight. Bb were added at a MOI = 10, centrifuged at 300 x g for 5 min to facilitate contact, and incubated at 37°C in 5% CO_2_. At the indicated times post-infection, total RNA was harvested from each triplicate well using an RNeasy® kit (Qiagen). Total RNA was reverse transcribed into cDNA using ImProm II® reverse transcriptase (Promega) per the manufacturer’s instructions. cDNA was quantified by real-time quantitative PCR (Q-PCR) using a Light Cycler (Roche) rapid fluorescence temperature cycler, as previously described [41]. The PCR primer sets were purchased through Integrated DNA Technologies and are listed in Table 1. Data are plotted as fold change over β-actin, where the values for the unstimulated wild-type control over β-actin were set = 1, and all other values were normalized to their own β-actin values and compared to the wild-type control. Similar experiments were performed at least three times.

**Table 1:** List of PCR primers used.

**Primer**	**Forward (5’ 3’)**	**Reverse (5’ 3’)**
**CXCL1 (KC)**	**GGG ATT CAC CTC AAG AAC ATC CAG**	**TTT CTG AAC CAA GGG AGC TTC AGG**
**CCL3 (MIP-1α)**	**CCG GAA TTC CGG AGA CTC TCA GGC ATT CAG TT**	**CGC GGA TCC GCG ATG AAG GTC TCC ACC ACT GC**
**CCL4 (MIP-1β)**	**CGC GGA TCC GCG ATG AAG CTC TGC GTG TCT GC**	**CCG GAA TTC CGG GCT GGA GCT GCT CAG TTC AA**
**CXCL2 (MIP-2α)**	**AGT GAA CTG CGC TGT CAA TG**	**GAG AGT GGC TAT GAC TTC TGT CTG**
**CCL2 (MCP-1)**	**AGC AGG TGT CCC AAA GAA GCT GTA**	**AAA GGT GCT GAA GAC CTT AGG GCA**
**CCL5 (RANTES)**	**GCC CAC GTC AAG GAG TAT TTC TAC**	**CTT GAA CCC ACT TCT TCT CTG G**
**β-actin**	**TGG AAT CCT GTG GCA TCC ATG AAA C**	**TAA AAC GCA GCT CAG TAA CAG TCC G**

### Immunofluorescence microscopy and phagocytosis assay

Naïve MØs and DCs were seeded in 12-well plates containing glass cover slips at 2 x 10^5^ cells per well in RPMI.B. For DCs, glass cover slips were coated with 0.01% poly-L-lysine (Sigma) for at least 24 hours prior to cell seeding to enhance cell adherence. Seeded APCs were either left untreated or were pre-treated overnight with live Bb N40 (MOI = 10) or recombinant IL-10 (2ng/ml; BD Pharmingen) before performing the assay. For the phagocytosis assay, green fluorescent protein (GFP)-producing Bb B31 were added to each well at a MOI = 10 and centrifuged at 300 x g for 3 min to facilitate Bb-APC contact. At the indicated times post-infection, supernatants were removed and the cells washed 2x in PBS to remove unbound Bb. APCs were then fixed in 4% paraformaldehyde overnight prior to immunochemical staining. To identify lysosomal compartments, fixed APCs were membrane-permeablized with 1% Triton-X-100 and stained with 2 μg/ml of a Lysosomal-associated membrane protein 1 (LAMP)-1-specific antibody (ID4B; Developemental Hybridoma, University of Iowa) and visualized with 2 μg/ml tetramethylrhodamineisothiocyanate (TRITC)-goat anti-rat antibody (Southern Biotech). 4',6-diamidino-2-phenylindole (DAPI; Fisher Scientific) was added at 250nM to visualize the nuclei. Cover slips containing the stained APCs were mounted onto glass slides using Fluoromount® (Southern Biotech) and examined by phase and immunofluorescence microscope (Leica DM IRB inverted microscope). Epifluorescent images of fields containing 75-150 APCs were captured at 200x magnification and overlayed on bright-field images using Q Capture® software (QImaging software); at least three separate fields were assessed for each slide. For quantitative analysis of phagocytosis, the percentage of APCs containing at least one internalized Bb particle was determined for each field (#APCs containing Bb/total number of APCs). This ratio was averaged for the total fields per triplicate slides in three independent experiments.

### Determination of reactive oxygen intermediates (ROI)

Naïve MØs and DCs were seeded in 96-well black plates with clear bottom (Costar) at 10^5^ cells per well in RPMI.B for 10 to 15h prior to Bb-stimulation. Immediately preceding stimulation, the RPMI.B media was washed to remove serum and APCs were pre-loaded with 10µM 2’, 7’-dichlorodihydrofluorescein-diacetate (DCFH-DA, i.e. DCF; Invitrogen) in serum-free HBSS for 15 minutes at 37°C before washing to remove the excess dye. These loaded APCs were incubated for an additional 30 min in fresh HBSS at 37°C in the absence or presence of the indicated agonists/antagonists prior to stimulation with Bb. Bb were then added at MOI = 10, centrifuged at 300 x g for 3 min to facilitate Bb-APC contact, and immediately assessed for fluorescence using a FLUOstar Omega microplate reader (BMG LABTECH). Some samples received the NADPH oxidase inhibitor DPI (10μM) to block the ROS response; 10μM was shown in preliminary experiments to completely suppress ROS responses without being toxic to cells, as assessed by trypan blue exclusion and MTT assay (data not shown). The relative fluorescent intensity units (RFU) and the change in RFU over time were acquired at 480nm excitation/520nm emission at 1 min intervals for 45 min.

### Determination of reactive nitrogen intermediates (RNI)

Naïve MØs and DCs were seeded on 24-well tissue-culture treated plates at 4 x 10^5^ cells per well in 0.5 ml of RPMI.B and allowed to adhere overnight. Bb were added at a MOI = 10, centrifuged at 300 x g for 5 min to facilitate contact, and incubated at 37°C in 5% CO_2_. At the indicated times post-infection, supernatants were collected and assessed for nitric oxide (NO) production using the Griess assay to determine nitrite (NO_2_) levels as described previously [49]. Briefly, equal volumes of 1% sulfanilamide in 30% acetic acid and 0.1% N-(1-napthyl) ethylenediamine dihydrochloride in 60% acetic acid were added to 50 μl of cell supernatants in a 96-well plate. NO_2_ levels were determined based on the absorbance values acquired at 570 nm wavelengths and compared to a NaNO_2_ standard using a Versamax® plate reader (Molecular Devices).

### Detection of APC surface molecules

Naïve MØs and DCs were seeded onto 6-well plates at 3 x 10^6^ cells/well in 1ml RPMI.B and allowed to adhere overnight. Bb were added at MOI = 10, centrifuged at 300 x g for 5 min to facilitate contact, and incubated at 37°C in 5% CO_2_. At the indicated times post-infection, the recovered cells were transferred to polystyrene tubes and incubated for 30 min with blocking buffer (PBS containing 1% BSA) along with 10 μg/ml anti-Fc receptor antibodies (BD Pharmingen) on ice. APCs were then incubated with either FITC- or PE-conjugated mAbs against cell surface markers CD11b, CD11c, MHC II, CD80, CD86, CD40, F4/80, or appropriate isotype controls (BD Pharmingen) at 5 μg/ml for 20 min on ice. APCs were then washed 3x with cold PBS, and fluorescent levels measured using a FACS Caliber® flow cytometer (BD Biosciences). Quantitative analysis of the flow cytometry data (e.g. mean fluorescent intensity and percent positive) was performed using Cellquest (BD Biosciences) and FlowJo (Tree Star, Inc.) software. 

### Statistical analyses

Statistical analyses were performed using InStat software (GraphPad Software). Based on these software determinations, the quantitative differences between sample groups were determined by either Kruskal-Wallis (non-parametric ANOVA) followed by Dunn’s Multiple Comparisons Test, parametric ANOVA followed by a Tukey Test, or two-tailed Student’s T test. P values of <0.05 were considered to be significant. 

## Results

### Bb elicit high levels of IL-10 production by both MØs and DCs that suppress cytokine responses

**Figure 2:**
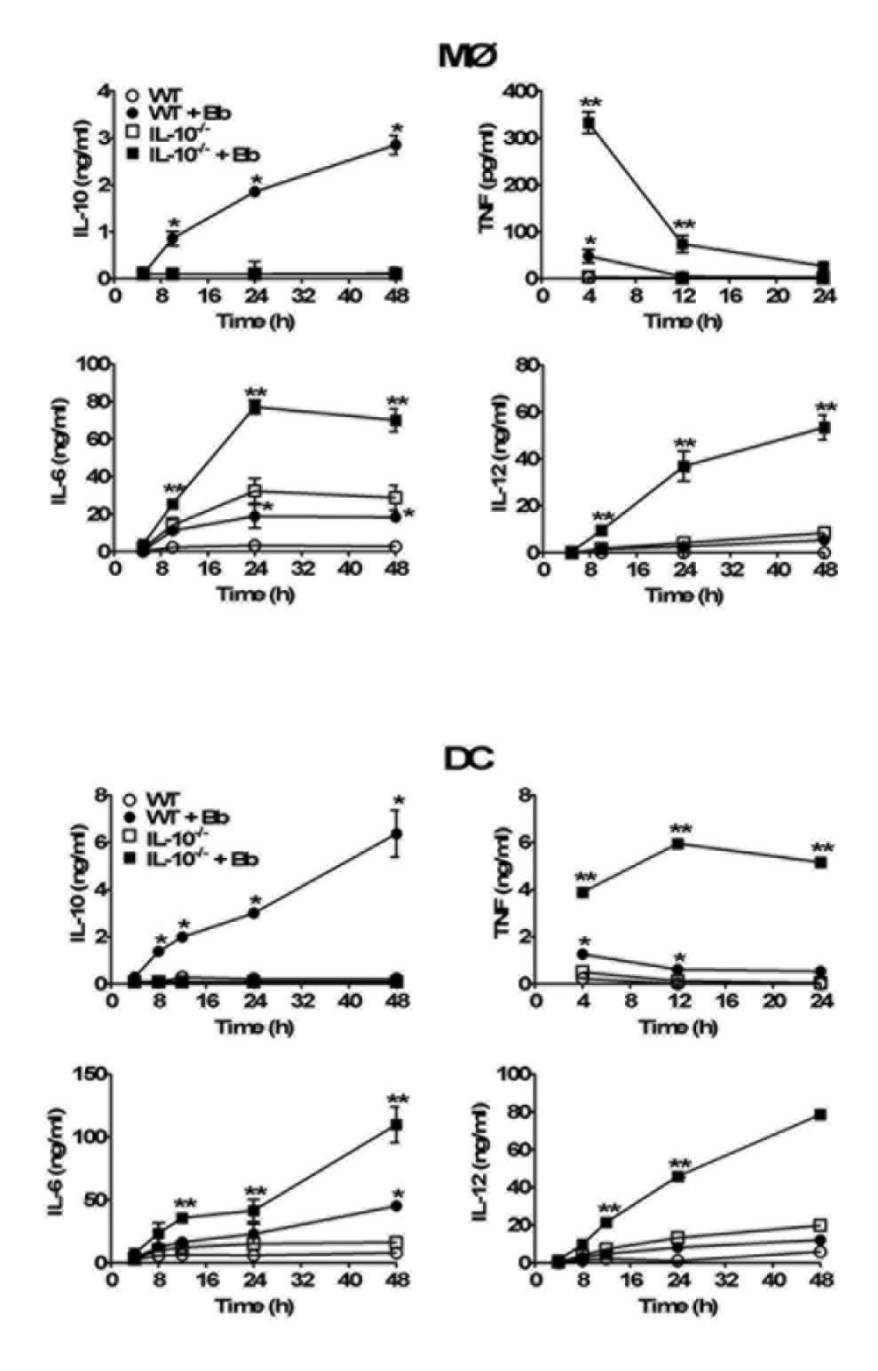
Effects of Bb-elicited IL-10 on the production of proinflammatory cytokines by MØs and DCs *in* *vitro*. MØs (upper panel) or DCs (lower panel) derived from either wild type (wt) C57BL/6 or IL-10^-/-^ mice were co-cultured with Bb (MOI=10) at 37C^o^. Culture supernatants were collected at the indicated times post-stimulation and cytokine content assessed by ELISA. Each symbol represents the average of triplicate samples from at least three separate experiments. Statistically significant (P<0.05) values are indicated between Bb-stimulated versus unstimulated APCs (*), or stimulated B6 versus IL-10^-/-^ APCs (**).

Kinetic studies were performed to assess the production of IL-10 by MØs and DCs, as well as its effect on production of pro-inflammatory cytokines. The addition of Bb to B6 MØs and DCs elicited significant levels of secreted IL-10 within 8h of simulation, as determined by ELISA (Figure 2). Intact Bb also elicited a variety of pro-inflammatory cytokines by both MØs and DCs with similar kinetics to IL-10, albeit at relatively low levels; these include IL-6, IL-12, and TNFα. To test whether the Bb-elicited IL-10 suppresses the production of pro-inflammatory cytokines from both APC types, parallel experiments were performed with MØs and DCs isolated from IL-10^-/-^ mice. As expected, IL-10^-/-^ APCs do not produce IL-10 in response to Bb, however, the production of all assessed proinflammatory cytokines were significantly higher than that produced by B6 APCs (Figure 2). 

**Figure 3:**
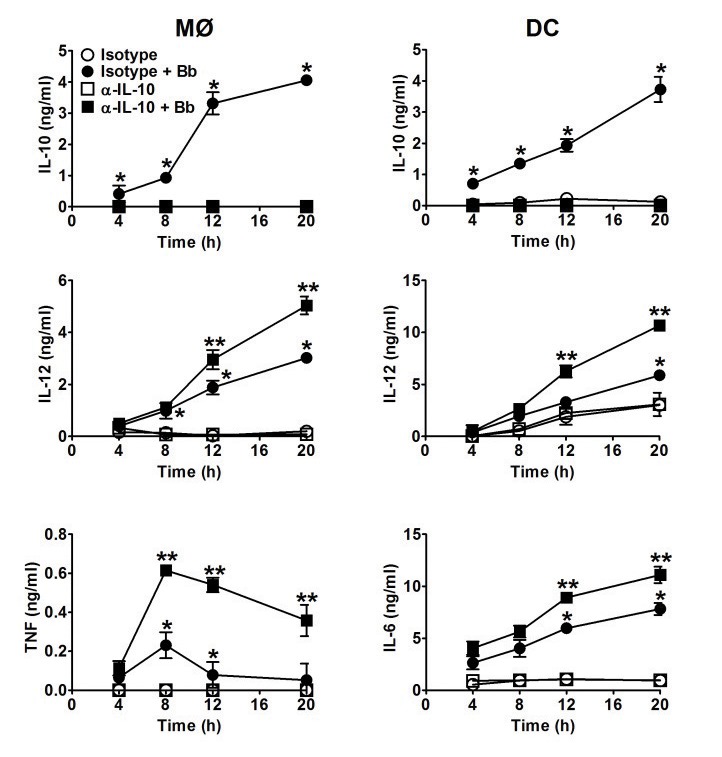
Use of IL-10-blocking antibodies to assess IL-10 effects on MØs and DC cytokines. Experiments were performed as in Figure 2, except MØs and DC from B6 mice were preincubated with 3μg/ml of either an IL-10-blocking (αIL-10) or isotype control antibody for 30 min before adding Bb (MOI=10) at 37°C. Each symbol represents the average of triplicate samples from at least three separate experiments. Statistically significant (P<0.05) values are indicated between Bb-stimulated versus unstimulated APCs (*), or stimulated isotype Ab-treated B6 versus αIL-10-treated APCs (**).

To confirm whether these findings were directly due to Bb-elicted IL-10 rather than effects inherent to IL-10-deficiency, parallel experiments were performed using B6 APCs stimulated with Bb in the presence of neutralizing antibodies against IL-10 (αIL-10). These antibodies were able to neutralize all the IL-10 produced *in vitro* by the APCs (Figure 3), and this blockage resulted in a similar increase in the production of pro-inflammatory cytokines after Bb co-culture as seen in the IL-10^-/-^ APCs (compare Figures 2 and 3). Together, these findings indicate that both MØs and DCs are major producers of Bb-elicited IL-10, and that these levels of IL-10 can act back on MØs and DCs to suppress the production of pro-inflammatory cytokines involved in inflammatory responses to Bb. 

### Chemokine production by MØs and DCs is suppressed by Bb-elicited IL-10

**Figure 4:**
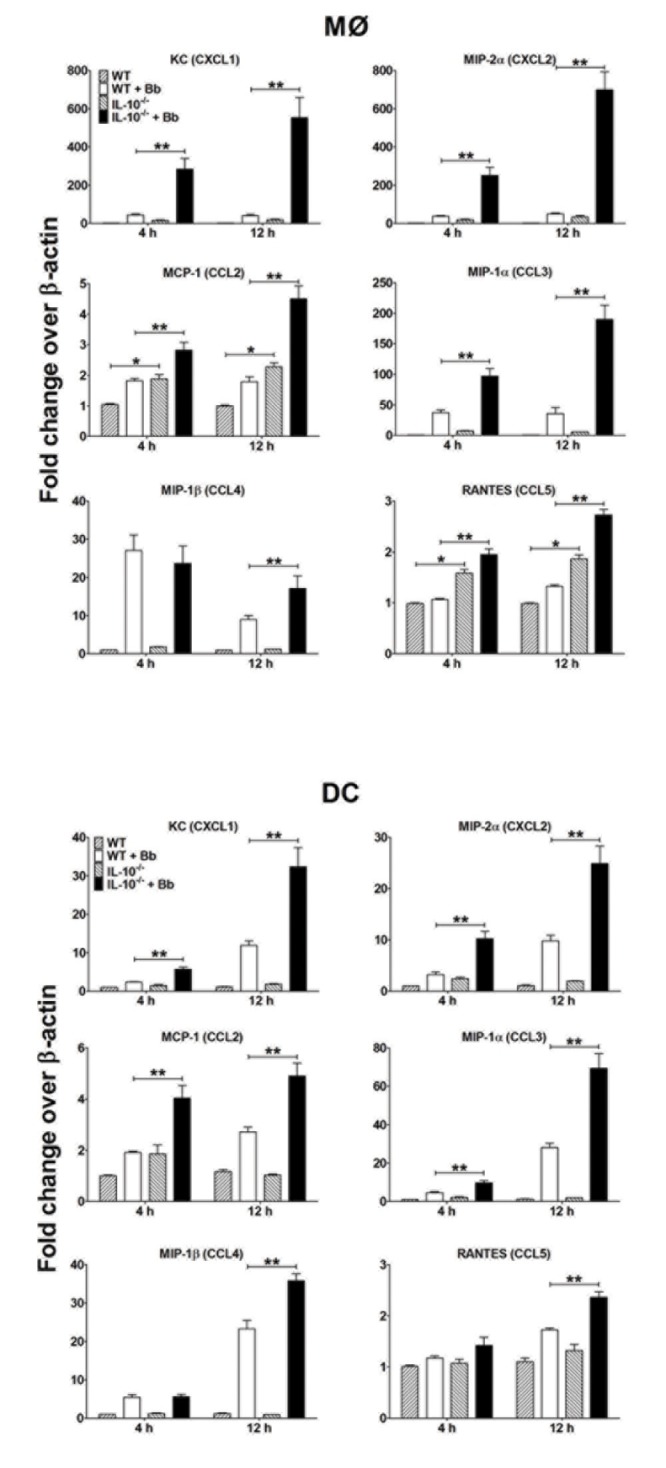
Effects of Bb-elicited IL-10 on chemokine expression by APCs: MØs (upper panel) and DCs (lower panel) from B6 and IL-10^-/-^ mice co-cultured with Bb at MOI=10 at 37°C. APCs were collected at the indicated times post-stimulation and total RNA was purified and reverse transcribed into cDNA for Q-PCR analyses. The transcript levels of each chemokine are normalized to levels of β-actin for each sample and reported as fold-induced over the baseline values for unstimulated MØs (upper panel) and DCs (lower panel). Each bar represents the average of triplicate samples from at least three separate experiments. Statistically significant values are indicated between unstimulated and Bb-stimulated APCs (*), or stimulated B6 versus IL-10^-/-^ APCs (**).

Neutrophil-recruiting chemokines such as CXCL1 (i.e. KC) are known to play critical roles during Bb infection [50,51]. Since MØs and DCs are major producers of chemokines, we assessed whether Bb-elicited IL-10 can down-regulate chemokine production by APCs. The chemokines assessed were chosen to sample those capable of attracting a range of leukocytes; CXCL1 (KC) and CXCL2 (GROβ) attract neutrophils, whereas CCL2 (MCP-1), CCL3 (MIP-1α), CCL4 (MIP-1β), and CCL5 (RANTES) are more directed towards monocyte/macrophages and T cells. Addition of Bb induced up-regulation of transcript levels for all chemokines assessed, including CXCL1, CXCL2, CCL2, CCL3, CCL4, and CCL5 by MØs and DCs as early as 4h post-infection (Figure 4). These transcript levels continued to increase over 12h post-stimulation in DCs, whereas transcript levels in MØs appeared relatively consistent over this timeframe. Addition of Bb to IL-10^-/-^ APCs displayed a significantly enhanced upregulation of many chemokines at 4h compared to B6 APC, including CXCL1, CXCL2, and CCL3, as well as CCL2 in DCs only. By 12h post-infection, all chemokines assessed in both IL-10^-/-^ APC types were upregulated compared to B6 cells, indicating that Bb-elicited IL-10 does suppress chemokine production by both MØs and DCs. 

### IL-10 inhibits Bb uptake and trafficking by MØs

**Figure 5:**
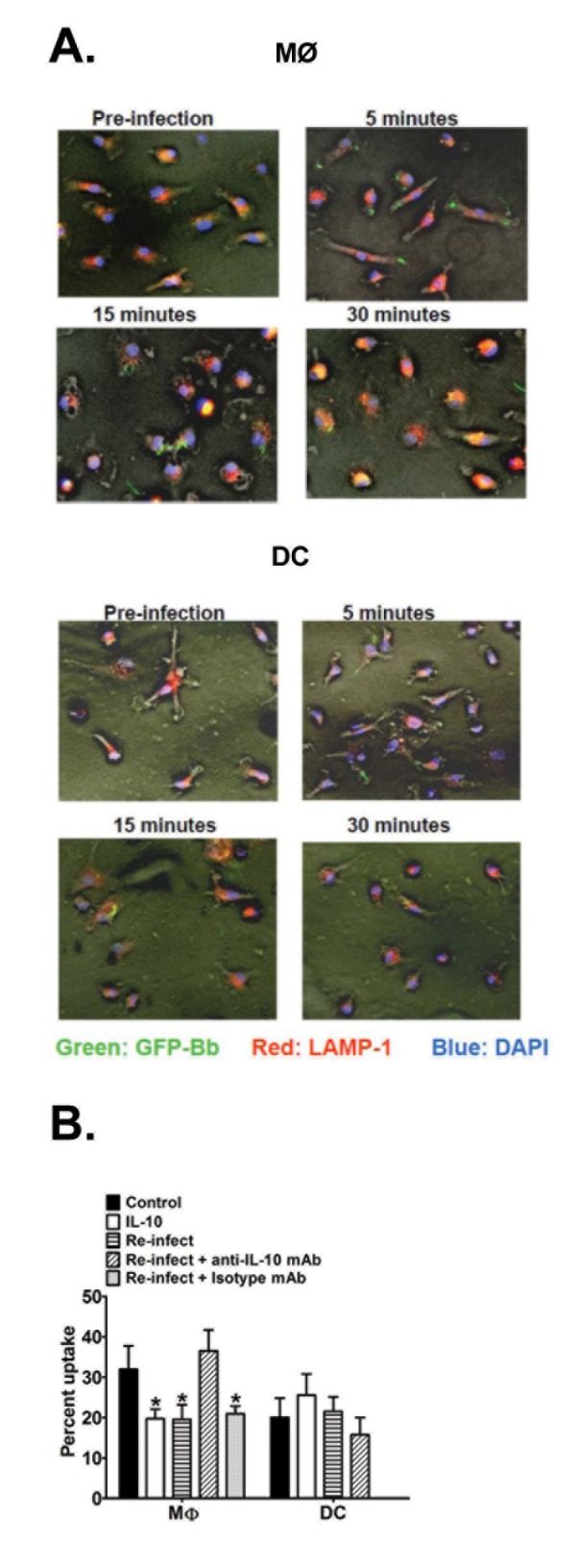
Effect of IL-10 on Bb uptake and trafficking by APCs. A. MØs (upper panel) and DCs (lower panel) from B6 mice were cultured on glass coverslips and infected with GFP-expressing (green) Bb (MOI=10) at 37°C. At 5, 15, and 30 min post-infection, APCs were fixed with 4% paraformaldehyde and permeablized for staining of LAMP-1 using TRITC-labeled (red) antibodies; nuclei are stained with DAPI (blue). Immunofluorescent images (200x) are representative of triplicate samples from three separate experiments, each with at least 3 different fields of views. (B) Quantitative analysis of Bb phagocytosis by APCs. MØs and DCs from B6 mice were infected with GFP-expressing Bb as in A (above) under five different conditions: GFP-Bb only (control), recombinant IL-10 (2ng/ml) administered overnight prior to GFP-Bb infection (rIL-10), infected with non-fluorescent Bb overnight prior to GFP-Bb infection (Re-infect), same as Re-infect, but initial infection was performed in the presence of anti-IL-10 antibody (Re-infect + anti-IL-10 mAb) or an isotype control (Re-infect + isotype mAb). Percent internalization was calculated as percentage of APCs per field of view containing internalized Bb at 30 min post-Bb infection. Data represents the average of ten separate fields of views, each containing 75-150 APCs, comprising at least three separate experiments. * Indicates statistically significant values (*P*≤0.05) compared to cells cultured with GFP-Bb only.

To assess whether Bb-elicited IL-10 directly affects phagocytosis of Bb by APCs, MØs and DCs were seeded onto glass coverslips, co-incubated with a GFP-expressing B31 strain of Bb, and images were captured at different times post-infection by immunofluorescence microscopy (IM). Bb intake by MØs occurred as early as 5 min post-infection, as demonstrated by the presence of tightly circularized Bb particles (green) within the APCs (Figure 5A, top panels), and these numbers were slightly increased at 15 and 30 min post-infection. Co-staining with LAMP-1-specific antibodies indicated that Bb were trafficked to LAMP-1-containing compartments within 15 min, and most internalized Bb showed co-localization in lysosomal compartments by 30 min. DCs showed a similar kinetics of Bb uptake and trafficking as MØs, though the total numbers appears to be less (Figure 5A, lower panels). Pre-treatment of MØs and DCs with cytochalasin D completely inhibited the internalization, as all Bb were observed in spirochetal form outside of APCs at all times post-infection (data not shown). 

To quantify whether IL-10 affects the uptake and trafficking of Bb by APCs, the percentages of cells containing internalized, “non-linear” Bb particles were compared between B6 APCs and those pretreated with a physiological level of rIL-10 (based on levels observed in Bb-stimulated APCs; Figure 2). At 30 min post-infection, MØs pre-treated with rIL-10 demonstrated a significant reduction in percent Bb intake as compared to control MØs (Figure 5B). As another physiological model, IL-10 was induced naturally by pre-treating MØs with non-fluorescent N40 Bb overnight before re-infecting the same MØs with the fluorescent GFP-B31 strain. This treatment produced a similar reduction in Bb intake as those treated with exogenous rIL-10. To delineate whether the suppression for the “re-infection group” is due to IL-10, neutralizing IL-10 antibody was added during the pretreatment with N40 Bb, which resulted in the uptake reverting back to the control MØs levels; pre-treatment with an isotype control antibody had not such effect. Parallel assays were performed to determine the effects of IL-10 on Bb phagocytosis by DCs (Figure 5B). Control DCs displayed a slightly lower rate of Bb uptake as observed by MØs, however the addition of rIL-10 or pretreatment with Bb did not appear to significantly affect Bb uptake and trafficking by DCs. These data suggest that Bb-elicited IL-10 suppresses phagocytosis of Bb by MØs, but not DCs.

### Bb-elicited IL-10 suppresses the production of reactive oxygen intermediates (ROI) and reactive nitrogen intermediates (RNI) by MØs in response to Bb

**Figure 6:**
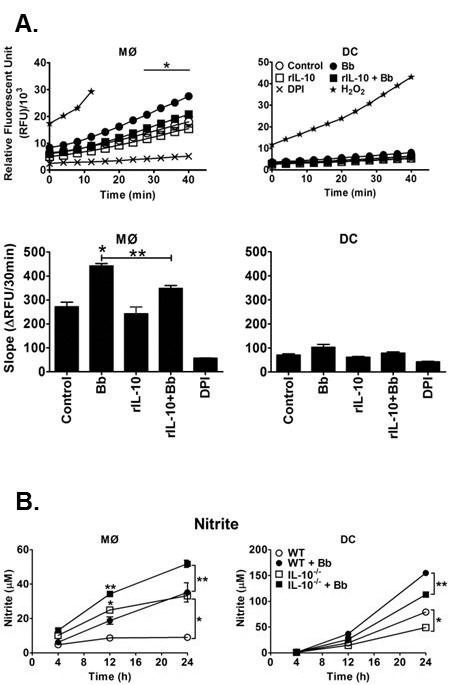
Effects of IL-10 on Bb-induced ROS and RNI production by APCs. A. MØs (left panels) and DCs (right panels) were cultured in 96-well plates for 15 h before loading with DCF for 15 min. Cells were then pre-treated with or without rIL-10 (2ng/10^5^ cells) or the NADPH oxidase inhibitor DPI (10μM) for an additional 30 min prior to Bb-stimulation; 0.01% H_2_O_2_ provided a positive control. ROS production was assessed by detecting the relative fluorescence intensity (RFU) values for up to 40 min post-stimulation. Top: Data are expressed as raw RFU values per min over a 40 min interval (Left: MØs, Right: DCs). Middle: Slope of RFU for each condition is calculated as changes in RFU over time for the first 30 min. Data are representative of three separate experiments. Statistically significant (P<0.05) values are indicated compared to unstimulated control (*) or Bb stimulation (**). B. MØs (left panels) and DCs (right panels) were stimulated with Bb for 24h. Supernatant nitric oxide (nitrite) levels were assessed using the Griess assay. Statistically significant (P<0.05) values are indicated compared to unstimulated controls (*) or Bb stimulation (**).

Live Bb and Bb components are capable of eliciting ROIs in phagocytic cells [25,52,53]. To test whether Bb-elicited ROI production in MØs and DCs is affected by IL-10, a DCF-based assay was employed to detect intracellular ROI levels in response to Bb. Co-culture of Bb with MØs caused a significant upregulation of ROI, as indicated by both the raw RFU units as well as the slope of change (Figure 6A). Pretreatment of MØs with a physiological amount of rIL-10 (1ng/10^5^ cells) before adding Bb caused a significant reduction in ROI compared to Bb treatment alone, and this suppressed response was similar to that seen in untreated MØs or cells treated with rIL-10 alone. MØs stimulated with Bb in the presence of the NADPH oxidase inhibitor, diphenylene iodonium (DPI), showed no increase in RFU values, indicating the ROS was internally generated. Parallel experiments using DCs indicated these cells were unable to elicit significant ROI in response to Bb. These data indicate that Bb-elicited IL-10 can inhibit ROI production by MØs exposed to Bb.

The effects of IL-10 on RNI production by MØs and DCs in response to viable Bb was assessed using the Griess assay. The addition of Bb to MØs caused a significant upregulation of nitric oxide at 24h post-stimulation (Figure 6B). While MØs from IL-10^-/-^ mice had higher inherent nitric oxide production compared to B6 MØs, addition of Bb to IL-10^-/-^ MØs did display a significant increase compared to Bb-stimulated B6 MØs at both 12 and 24h post-stimulation. Parallel experiments using DCs showed that Bb co-culture produced a significant upregulation of NO at 24h post-stimulation compared to control DCs (Figure 6B), similar to the trend observed with MØs. Conversely, IL-10^-/-^ DCs displayed lower nitric oxide production compared to B6 both in the presence and absence of Bb. These data indicate that Bb-elicited IL-10 can suppress RNI responses by MØs, but not DCs, in response to Bb stimulation.

### Bb in the presence/absence of IL-10 has varied effects on regulating co-stimulatory molecules on the surface of APCs

**Figure 7:**
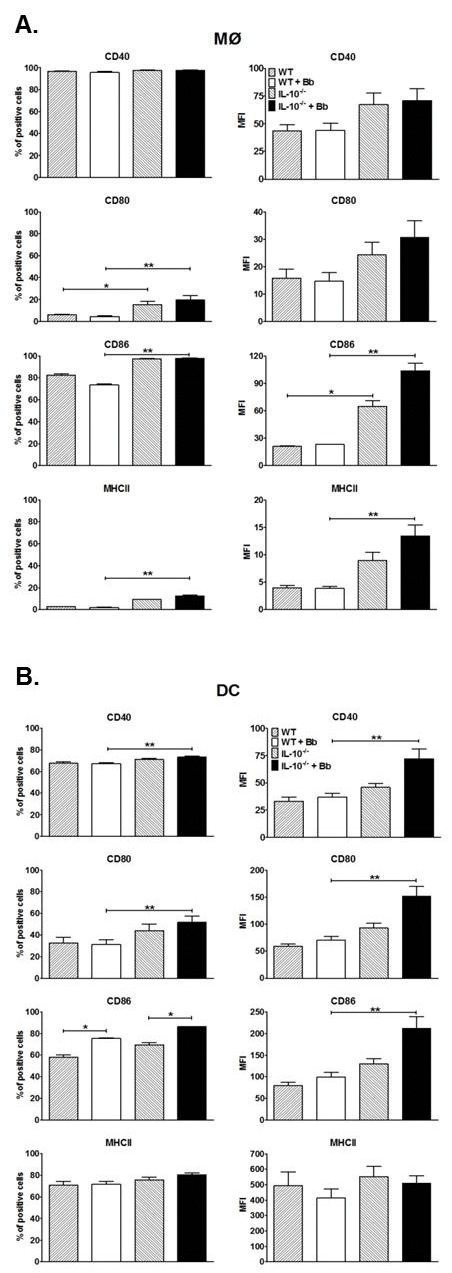
Effect of IL-10 on Bb-elicited upregulation of surface co-stimulation molecule expression on APCs. MØs (A) and DCs (B) expanded from B6 and IL-10^-/-^ mice were cultured with Bb for 24h before staining with fluorescence antibodies specific for the indicated surface molecules and analyzed by flow cytometry. Data are reported both as percentage of positive cells (left panels) and mean fluorescent intensity (MFI). Each bar represents triplicate samples from at least three separate experiments. Statistically significant (P<0.05) values are indicated compared to unstimulated (*) or Bb-stimulated APCs (**).

Another important function of MØs and DCs are to serve as APCs to drive T cell activation in response to pathogens. Because expression of certain co-stimulatory surface receptors are central to APC activation of T cells, the effects of Bb stimulation and Bb-elicited IL-10 on expression of a subset of these receptors was assessed by flow cytometry. All MØs appears to express CD40 and Bb stimulation did not change the density of this receptor in the presence or absence of IL-10 (Figure 7A). The percentage of MØs expressing CD80, CD86, and MHC class II did not change after co-culture with Bb, though the absence of IL-10 appeared to increase the percentage of cells expressing CD80 independent of Bb stimulation. Assessment of IL-10^-/-^ MØs indicated that Bb-elicited IL-10 had no effect on the density of CD80 expression after Bb activation, however it did downregulate the density of CD86 and MHC II, though the absence of IL-10 did appear to upregulate CD86 levels irrespective of Bb stimulation. Overall, these findings suggest that MØs do not upregulate any of the assessed co-stimulatory surface receptors in response to Bb, and some of these reduced responses appear due to the presence of Bb-elicited IL-10.

 Assessment of DCs indicated that Bb co-culture increased the percentage of cells expressing CD86, but had no effect on the percentage of cells expressing the other assessed surface receptors (Figure 7B). Bb stimulation alone also had no effect on the density of all of the assessed co-stimulatory receptors on DCs. Assessment of IL-10^-/-^ DCs indicated that Bb-elicited IL-10 can suppress the percentage of cells expressing CD40 and CD80, but had little effect on the percentage of cells expressing CD86 and MHC II compared to Bb stimulation alone. Bb-elicited IL-10 did significantly suppress the density of CD40, CD80, and CD86 expressed on Bb-stimulated DCs, but had no effect on MHC II density. Overall, these findings suggest that DCs only upregulate CD86 among all of the co-stimulatory receptors assessed in response to Bb stimulation, and many of these reduced responses appears due to the presence of Bb-elicited IL-10. 

## Discussion

Bb is an obligate parasite whose natural infection cycle requires it to persist in an immunocompetent host for an extended time post-infection. This pathogen largely resides within the extracellular matrix of multiple tissues and appropriate innate responses appear critical for controlling infection. MØs and neutrophils appear able to phagocytose and clear Bb *in vitro*, however the ID_50_ is ≤50 organisms *in vivo*, suggesting their responses are much less efficient within host tissues. Previous work in our lab and others [35,36] indicates that Bb causes a rapid and substantial increase in IL-10 production by MØs, and that IL-10 adversely affects Bb clearance *in vivo* mainly via inhibition of innate immune responses [37,41]. Based on these findings, we speculate that certain skin resident APC’s are the major producer of Bb-elicited IL-10, which then act to suppress critical immune properties of these resident cells that are necessary to initiate and modulate an effective immune response. Our goal was to assess the relative abilities of MØs and DCs to elicit IL-10 in response to Bb, and to delineate any detrimental effects this IL-10 has on their ability to phagocytose/traffic Bb, as well as to subsequently produce inflammatory mediators and express surface co-stimulation markers that are associated with effective clearance of pathogenic bacteria.

In this study, bone marrow-derived MØs and DCs displayed rapid IL-10 production in response to Bb, with levels apparent between 4-8 post-infection, and which continued to increase throughout the 48h period they were assessed. The kinetics of IL-10 production was similar between MØs and DCs, though DCs appeared to produce ~2-fold higher levels at all times assessed. These kinetics were similar to those previously reported by our group and others for MØs *in vitro* [41,42,54,55], and correlate with the rapid IL-10 increase seen in the skin of infected mice [42]. Studies by Sonderegger et al have reported that MØs and CD4^+^ T cells are the major producers of Bb-elicited IL-10 in infected mouse joints, though these levels were assessed at ≥2 weeks post-infection [40]. However, it is unlikely that CD4^+^ T cells would be involved in the increased IL-10 levels seen in infected skin 24-48h post-infection by previously naïve mice, particularly with the well-documented delay in adaptive immune response to Bb infection, as there would be insufficient time for the development of Bb-specific T cells [7]. Based on our described kinetics of IL-10 production and their presence as skin-resident cells, it is likely that MØs and DCs are a significant source of Bb-elicited IL-10 at these early times post-infection.

To address whether the IL-10 elicited by these skin-resident APCs might influence the ability of adjacent cells to recognize and kill Bb, assays were performed to assess phagocytosis characteristics and production of mediators associated with bacterial killing. In these studies, Bb were taken up by MØs and DCs as early as 5 min post-infection, packaged relatively tightly into phagosomal compartments, and localized to the vicinity of LAMP-1 containing compartments as early as 15-30 min after intake, which was similar to other published studies assessing phagocyte clearance of Bb [22,56-58]. The effects of Bb-elicited IL-10 on phagocytosis was tested in two ways: 1) pretreatment with physiologic doses of rIL-10 (based on Figure 2) and 2) pretreatment with non-fluorescent Bb to elicit natural production of IL-10. In each of these cases, the trafficking of Bb to LAMP-1-containing compartments by MØs was significantly suppressed by 30 min post-infection, and the suppression was lost in the presence of IL-10-blocking antibodies, confirming that the effects were IL-10-specific. Thus, Bb-elicited IL-10 appears to have a direct effect on phagocytosis and trafficking of Bb by MØs, and presumably affects the killing efficiency during clearance. Alternatively, uptake and clearance by BMDCs appeared to be relatively unaffected by IL-10. This could be a reflection of the somewhat lower phagocytosis rate exhibited by DCs compared to MØs, but more likely reflects a general mechanistic difference between MØs and DCs in terms of Bb retention and killing (see discussion below).

The production of various ROIs and RNIs, such as superoxide (O_2_^-^), hydrogen peroxide (H_2_O_2_), hydroxyl radicals (OH^.^), and nitric oxide (NO), during activation of phagocytes are essential to the killing of many bacterial pathogens [59-61]. There have been reports that IL-10 can affect the generation of ROI and RNI in response to certain pathogens [62-64], but little is known regarding Bb. In the current studies, Bb elicited significant production of ROI and RNI from MØs, similar to results previously reported by our lab and others [21,65,66], and also elicited RNI from DCs, which is previously unreported for Bb. However, introduction of physiological levels of IL-10 significantly reduced the production of both ROI and NO by MØs. Alternatively, while DCs showed increased production of NO, but not ROI, in response to Bb, these IL-10 levels were unable to suppress the relatively low levels produced by DCs. Whereas MØs are well-known for their ability to phagocytose and kill pathogens, largely through RNI, ROS, and other phagosomal mediators, “classical” dendritic cells are more known for their ability to phagocytose pathogens and subsequently present antigens to T cells [61,67,68]. It is possible that the differences seen on IL-10 effects between MØs and DCs may reflect the generally lessened production of RNI and ROS by DCs compared to MØs. More directed studies to determine if these effects are common to all of the different DCs subsets that have recently been identified are beyond the scope of this study [69,70]. The importance of ROI and NO in controlling Lyme disease is unclear. Infection of mice that lack functional NADPH oxidase or iNOS determined they possess similar Bb levels and arthritis development as control mice [65,71,72]. However, ROI and RNI can damage various Bb components, such as lipoproteins [63,64] and proteins containing free or zinc-containing cysteine thiols [73]. Bb does express SOD, which is required for Bb survival in the host [74]. Notably, Bb also lacks both free iron and iron-containing enzymes, suggesting they may be less susceptible to ROS-mediated damage [75]. Thus, while Bb appears well-adapted to evade damage via ROS and RNI-mediated mechanisms, the suppressive effects conferred by Bb-elicited IL-10 would add to this resistance. 

 Activation of MØs and other skin-resident immune cells by pathogens usually leads to production of proinflammatory mediators that act to recruit particular immune cells from the vasculature and activate them in the vicinity of these infectious agents. These events are capable of clearing most bacteria, but must eventually be shut down before extended exposure to these toxic immune mediators can damage host tissues; this is often mediated through production of anti-inflammatory mediators, of which IL-10 is one of the best-described. In these studies, both Bb-stimulated MØs and DCs secrete prototypic proinflammatory cytokines, such as TNFα, IL-6, and IL-12, within 6h of exposure. However, the levels appear to be substantially suppressed by Bb-elicited IL-10, since the levels of proinflammatory cytokines were significantly higher from MØs and DCs from IL-10^-/-^ mice or those where IL-10 was blocked by incubation with IL-10-specific antibodies. Similarly, a number of chemokines known to recruit leukocytes to infection sites were upregulated by MØs and DCs during Bb infection [76], but many of these were suppressed in the presence of Bb-elicited IL-10, particularly the neutrophil-recruiting chemokines such as KC (CXCL1) and MIP-2α (CXCL2). While most of the sequella associated with Lyme disease are believed due to inflammatory responses against Bb or their associated agonists (e.g. lipoproteins, glycolipids, etc.) [18,21,23,77-82], there have been several reports of dysregulated immune responses during the early development of Lyme disease. Notably, neutrophils are observed to migrate into skin tissues containing Bb within hours of infection, but these numbers are not maintained and few neutrophils are observed within 1-3 days of infection [50,83], even though Bb numbers remain high in those tissues. The detrimental nature of this defect was noted by engineering Bb that constitutively produce the chemokine KC, and infections with this strain subsequently produce a more sustained recruitment of neutrophils to the infection site, resulting in enhanced Bb clearance [50]. The kinetics of the abbreviated neutrophil recruitment corresponds with the significant IL-10 production seen in skin tissues by 24h post-infection [42], and our findings demonstrating subsequent decreases in proinflammatory cytokines and neutrophil-attracting chemokines when IL-10 is blocked. Thus, Bb-elicited IL-10 may play a major role in the dysregulated inflammatory response observed early in the development in Lyme disease and may increase the chances of Bb to evade clearance by the cellular responses and subsequent dissemination to distant tissues. 

In addition to phagocytosing pathogens and mediating inflammatory responses, skin-resident APCs play a major role in bridging the innate and adaptive immune responses by the presentation of antigens on MHC class II molecules to T cells, as well as surface expression of co-stimulatory molecules whose ligation is required to allow optimal activation of T cells, such as CD40 and the B7 family molecules CD80 and CD86 [84-86]. In our studies, co-culture of MØs with Bb resulted in no upregulation of MHC II or any of the co-stimulatory molecules assessed, but blocking Bb-elicited IL-10 did allow subsequent upregulation of MHC II and CD86. When assessing DCs, co-culture with Bb had little effect other than an increase in the percentage of cells expressing CD86. However, removal of IL-10 allowed significant upregulation of all co-stimulatory molecules assessed. One of the hallmarks of Lyme disease is that, while infected animals eventually generate a robust and diverse antibody response, the development of this response appears to be delayed [7]. While these well-developed antibodies can prevent infection of naïve mice if administered co-currently with Bb, they cannot confer protection if the infection has been established for even a few days [6,87], suggesting that even briefly delayed development of the antibody response can promote establishment of Bb infection. Others have also shown that upregulation of co-stimulatory molecules by APCs in response to Bb is inadequate [81], and that the involvement of certain of these molecules (e.g. CD86) are needed for an optimal antibody response to Bb [88]. Together, these experiments suggest that the suppression of these co-stimulatory molecules on APCs by Bb-elicited IL-10 may contribute to the dysregulated adaptive responses generated during the development of Lyme disease.

Collectively, our findings indicate that both MØs and DCs are rapid and potent producers of IL-10 in response to Bb exposure. The IL-10 levels appear able to block many of the immune functions of these APCs that should be critical for controlling Bb infection, including phagocytosis/trafficking, induction of ROS and NO, secretion of inflammatory cytokines/chemokines, and upregulation of surface co-stimulatory molecules required for optimal activation of Bb-specific adaptive responses. Because MØs and DCs are believed to be largely responsible for moderating the early immune responses against Bb deposited into the skin, these findings suggest this IL-10 elicitation may be largely responsible for the dysregulated early leukocyte responses and delayed adaptive responses that are believed to have a major influence in the ability of Bb to efficiently disseminate and persist within susceptible hosts, as already suggested by the significantly enhanced Bb clearance and Bb-specific antibody responses displayed by IL-10^-/-^ mice [37,41]. Further studies are needed to determine whether treatments aimed at blocking these IL-10 effects can promote efficient clearance of these bacteria in both acute and chronic cases of Lyme disease.
