# Correction: *Borrelia burgdorferi* Elicited-IL-10 Suppresses the Production of Inflammatory Mediators, Phagocytosis, and Expression of Co-Stimulatory Receptors by Murine Macrophages and/or Dendritic Cells

**DOI:** 10.1371/annotation/680090aa-3e1b-4135-94d6-8082c09180d4

**Published:** 2014-01-14

**Authors:** Yutein Chung, Nan Zhang, R. Mark Wooten

There was an error in the affiliations for the second author. The current address indicated for Nan Zhang instead belongs to the first author, Yutein Chung.

