# Correction: *Borrelia burgdorferi* Elicited-IL-10 Suppresses the Production of Inflammatory Mediators, Phagocytosis, and Expression of Co-Stimulatory Receptors by Murine Macrophages and/or Dendritic Cells

**DOI:** 10.1371/annotation/2ce59bc4-fcf0-498f-86f0-376432428bf4

**Published:** 2014-01-28

**Authors:** Yutein Chung, Nan Zhang, R. Mark Wooten

The article has been republished on January 23, 2014 to correct the poor readability of Figures 1 through 7 in the original edition of the article, which can be viewed here: http://www.plosone.org/corrections/pone.0084980.001.cn.pdf

